# Sex-biased sound symbolism in French first names

**DOI:** 10.1017/ehs.2019.7

**Published:** 2019-07-12

**Authors:** Alexandre Suire, Alba Bossoms Mesa, Michel Raymond, Melissa Barkat-Defradas

**Affiliations:** ISEM, University of Montpellier, CNRS, EPHE, IRD, Montpellier, France

**Keywords:** Sound symbolism, first names, femininity, masculinity, voice

## Abstract

**Summary:**

Low- and high-frequency vowels in the stressed syllable of French first names may respectively project impressions of largeness/masculinity and smallness/femininity.

**Abstract:**

Given that first names can have a lifelong impact on the bearer, parents should choose a name based on the impressions they want their offspring to evoke in other people. This name-to-mental-image association can be mediated through sound symbolism: a natural link between the sounds and meaning of a word. From an evolutionary perspective, parents should pick names which sounds convey traits advantageous in human sexual selection: largeness and masculinity for males through lower-frequency sounds as opposed to smallness and femininity for females through higher-frequency sounds. Using a database of French first names from 1900 to 2009, we observed a sex-biased sound symbolism pattern in the last syllable, which is the perceptually prominent one in French. Male names were more likely to include lower-frequency vowels (e.g. /o/, /ã/) and female names higher-frequency vowels (e.g. /i/, /e/). Unexpected patterns in consonants were observed in masculine names with higher-frequency sounds (e.g. /s/, /ʃ/) in the last syllable and lower-frequency sounds (e.g. /b/, /g/) in the first syllable. However, little variance was explained and the modest size effect suggests that cultural traits influence these sex differences. Lastly, exploratory analyses revealed a phonetic masculinization in women's first names that has increased since the 1960s.

## Graphical abstract

**Figure:**
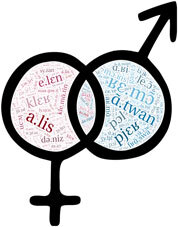

## Introduction

Arbitrariness, the notion that the sound and the meaning of a word are independent, has long been considered one of the most widely shared principles in linguistics. However, a growing body of evidence challenges this view, stating that there is a natural link between the sound units of a word – known as phonemes – and the mental image they evoke (see Svantesson 2017 for an overview). This principle, referred to as sound symbolism, is well illustrated by the ‘kiki-bouba’ and ‘maluma-takete’ experiments, in which participants are asked to associate such non-words to two figures of different shapes: results show above-chance matchings of ‘bouba’ and ‘maluma’ with a round silhouette, and ‘kiki’ and ‘takete’ with a sharp one (Ramachandran and Hubbard 2001; Werner 1957; Köhler 1947). Although it is uncertain to generalize the ‘kiki–bouba’ effect across cultures (see Bremner et al. 2013 and Cuskley et al. 2017), other similar sound-meaning mappings have been recorded in thousands of the world's languages, suggesting an underlying universal cognitive association mechanism (Blasi et al. 2016). Sexual selection for body size offers one possible explanation for why sound symbolism might be so ubiquitously distributed.

The first clue was provided by the ‘motivation-structural rules’ theory (Morton 1977), after observing that many animals modulate their vocalizations during competitive encounters: they use low-pitched vocalizations when their intention is to be threatening and dominant, and high-pitched vocalizations if they wish to appear conciliatory or submissive. The hypothesized reason is that the frequency of vocalizations reflects a projection of the individual's body size, a key determinant in the outcome of physical contests but also courtship interactions (Bradbury and Vehrencamp 2011). This notion was then extended to humans in the ‘frequency-code’ theory (Ohala 1984), which provides a plausible explanation for the observed vocal dimorphism in human voices. Before puberty, boys and girls exhibit similar vocal frequencies, until males experience a significant enlargement of their larynx and vocal folds under the influence of androgens, which lowers their vocal pitch and resonant frequencies to the point that they practically do not overlap with those of adult females (Titze 1989). Such findings hint towards the action of sexual selection and can be interpreted as a result of different selective pressures acting on each sex (Puts 2010). In males, lower-frequency voices could have been favoured within intra-sexual contests because they are perceptually associated with largeness (Pisanski et al. 2014; Xu et al. 2013; Pisanski and Rendall 2011; Rendall et al. 2007; van Dommelen and Moxness 1995), more masculine and more socially and physically dominant men (Hodges-Simeon et al. 2014; Puts et al. 2006, 2007; Xu et al. 2013; although see Armstrong et al. 2019 for why voice pitch may not be an honest signal of dominance). In contrast, higher frequencies in female voices could have been selected in mate-choice dynamics as such frequencies were shown to be associated with perceived smallness, femininity and more attractive women (Xu et al. 2013; Fraccaro et al. 2011; Puts et al. 2011; Jones et al. 2010; Feinberg et al. 2008; Collins and Missing 2003).

Although naming practices are assumed to be highly driven by sociocultural factors, few studies have underpinned the ultimate causes that have driven most male and female names to not overlap phonetically (Pitcher et al. 2013). As observed for other dimorphic traits in humans such as the body size and stature (Geary 1998; Puts 2010), one can reasonably assume that these two different sexual selective pressures on human voices could have driven the attested sexual phonetic dimorphism. Preliminary evidence has shown that across languages as diverse as English, Japanese, Chinese, Korean and several Native American and Australian languages, high- and low-frequency vowels are respectively associated with perceived smallness and largeness (Haynie et al. 2014; Shinohara and Kawahara 2010; Ultan 1978; Newman 1933; Sapir 1929), as well as perceived femininity and masculinity (Wu et al. 2013; Klink 2000). Thus, indexical cues that are known to be relevant to human mating (e.g. body size, masculinity and femininity) may be conveyed or projected in first names through sound symbolism, using an array of different phonemes that can differ in their intrinsic fundamental frequency (i.e. the perceptual correlate of pitch), formant frequencies (i.e. resonances of the vocal tract) and their dispersion (i.e. a proxy of the vocal tract length) (Knoeferle et al. 2017; Ohala 1994; Ultan 1978).

Although parents may not volitionally seek a large or small, dominant and attractive sounding name for their offspring, they might display an unconscious preference for either a more masculine or feminine name to suit their child's sex. This behaviour can be explained by the fact that gendered naming is an important tool of categorization in humans. Indeed, sex is one the most pervasive characteristic individuals first infer when interacting with others: distinguishing it by using different phonetic material for first names may find benefits in that it increases cognitive efficiency by allowing individuals to rapidly infer properties of sex category, even with little or no first-hand experience with that person. In turn, it enables individuals to tailor their expectations about the behaviours and capacities linked to the biological composition of that individual. Additionally, masculine and feminine names take on great importance in the reinforcement of an individual's sexual identity and gender role (Pilcher 2016). Although first names are not inherited and no studies have yet tackled the issue of their influence on reproductive success, it has been reported that first names can impact their bearers on several aspects: their physical perception (Zwebner et al. 2017; Hartung 2018; Perfors 2004; Erwin 1993; Hassebrauck 1988; Hensley and Spencer 1985), inferences on personality (Mehrabian 2001; Mehrabian and Piercy 1993; Leirer et al. 1982), attitudes and behaviours (Figlio 2007; Pelham et al. 2002), social desirability (Gebauer et al. 2012; Busse and Seraydarian 1978) and social outcomes (Cotton et al. 2008; Figlio 2005; Hodson and Olson 2005; Harari and McDavid 1973). Thus, it can be suggested that this cognitive bias could interfere during the naming process, since the phonetic peculiarities of forenames may underline and reinforce the perceptual associations of the biological and social characteristics linked to each sex through sound symbolism, which ultimately might be relatively important towards competitors and potential mates. Furthermore, to our knowledge, no societies (industrialized or not) currently use, or have been using, the same set of names for males and females. Lastly, it is worth noting that even though cultural evolution drives popularity and the emergence of novel names (e.g. Berger et al. 2012), it merely explains why individuals primarily perceive them as either male or female.

Sound symbolism has already been observed in the phonetic composition of English first names (Sidhu and Pexman 2015; Pitcher et al. 2013; Cassidy et al. 1999; Cutler et al. 1990). So far, only one study has formally tested these evolutionary hypotheses through the lens of sexual selection using a database of the thousand most popular English, American and Australian first names between 2001 and 2010 (Pitcher et al. 2013). In accordance with the evolutionary predictions, high-frequency vowels such as /i/ or /e/ were mostly attested in female names and low-frequency ones such as /u/ or /o/ in male names. Such differences were found on the first syllable, where stress is generally located and which is consequently perceptually prominent in English. However, the authors did not investigate consonant patterns nor take a look on the last syllable to ensure that no phonetic dimorphism was also present there.

The goal of the present study is to quantify the hypothesized phonetic dimorphism of male and female names, using a large sample size of popular first names in France that extends over the last century. In this context, this study extends the results that have already been observed in English first names. However, two major differences exist between French and English. First the lexical stress falls on the last syllable in French and most of the time on the first syllable in English. Secondly, all phonological units are not equally represented in French and English. For example, nasal vowels are attested in the former but absent in the latter. Moreover, analyses can be expanded by including consonants, for which patterns of sound symbolism have been previously reported (Nielsen and Rendall 2013; Maurer et al. 2006). Consequently, we expect to find sex-bias sound symbolic patterns in the phonemes of the stressed syllable in French names, namely back and nasal vowels and voiced consonants in male names, as they are produced at lower frequencies, as opposed to front vowels as well as voiceless consonants in female names, since their articulation produces noise in relatively higher frequencies (Knoeferle et al. 2017; Ohala 1994; Ultan 1978). Lastly, we will conduct exploratory analyses of the temporal variations of these sound symbolic patterns from 1900 to 2009 in order to examine whether they have remained constant or have evolved over time for each sex.

## Material and methods

### Data pre-treatment

Data was retrieved on September 2014 from the Institut National de la Statistique et des Études Économiques. We selected the most popular 100 female and 100 male names for each decade, ranging from 1900–1909 to 2000–2009. In order to control for population size, popularity was estimated by calculating the annual ranking position of each name and adding these up per decade. Although this approach excludes rare names, it captures naming practices properly for a given decade (Pitcher et al. 2013).

All retrieved names were subsequently transcribed independently by two native French-speaking phoneticians, following the International Phonetic Alphabet principles. When no agreement arose for certain transcriptions or when pronunciation was unknown, different web sources were used (e.g. https://fr.wiktionary.org/wiki). For each syllable of a name, we recorded the following articulatory features:

- The vowel place of articulation, which corresponds to the position of the tongue in the oral cavity during its articulation. As the tongue is closer to the lips, the sounds produced have an overall higher frequency spectrum (i.e. front vowels such as /i/). Conversely, sounds that are produced with the tongue retracted at the back of the mouth (i.e. back vowels such as /u/) have an overall lower spectral distribution. Central vowels (i.e. /a/) correspond to a position where the tongue is placed in the middle of the mouth. Acoustically, vocalic frontness and/or backness correspond to the frequencies of the second formant (i.e. the spectral peaks of the sound spectrum). The vowel height, which corresponds to the degree of aperture of the mandible (i.e. the open/close dimension, corresponding acoustically to the first formant), was not retained here, as it would produce redundant information with vowel articulation (i.e. multicollinearity in the statistical analyses).
- The vowel's nasality, which is determined by the low position of the velum during articulation, leads the air to flow through the nose as well as the mouth. This extra resonance, which results from the intervention of the nasal cavity during phonation, lowers the frequency of the sound in comparison to its non-nasal counterpart. Note that only one type of vowel (oral or nasal) can be found in each syllable.
- The consonant's manner of articulation, which is determined by the way the airflow escapes from the vocal tract during articulation. Here, we focused on plosives, which are produced by a complete closing of the airflow that causes its blocking before the air is suddenly released. This type of sound produces a burst noise that is typical of consonantal stops. We also focused on fricatives, which are produced with a major constriction of the airflow, which acoustically causes a turbulent noise. Owing to their manner of articulation, plosives generally produce lower frequencies than fricatives.
- The consonant's voicing, which is determined by whether the vocal folds vibrate or not during articulation. This new source of laryngeal noise explains why voiced consonants are lower in frequencies than voiceless ones.

All phonemes coded with examples of first names are given in Table 1.

**Table 1.** Examples of first names for each phoneme investigated (underlined)

Type of phoneme	Phonemes	Frequency domain	Name examples
Front vowels	/i/, /y/, /e/, /ø/, /ε/	High	Marie, Luc, Cécile, Eugène, Odette
Central vowels	/a/, /ə/	Central	Jeanne, Denise
Back vowels	/u/, /o/, /ɔ/	Low	Lou, Renaud, Paul
Nasal vowels	/ã/, /ε̃/, /ɔ̃/	Low	Antoine, Sylvain, Raymond
Voiced plosives	/b/, /d/, /g/	Low (voicing) Low (manner of articulation)	Norbert, Claude, Guy
Voiced fricatives	/ʒ/, /v/, /ʁ/, /z/	Low (voicing) High (manner of articulation)	Jean, Valérie, Suzanne, Claire
Voiceless plosives	/p/, /t/, /k/	High (unvoiced) Low (manner of articulation)	Pierre, Thibault, Nicolas
Voiceless fricatives	/ʃ/, /f/, /s/	High (unvoiced) High (manner of articulation)	Charlotte, Fabrice, Solange

### Statistical analyses

#### Analysis on sound symbolism

The aim of this analysis is to study and quantify sex differences in first names’ phonetic composition. According to our predictions, we expect to find in the stressed syllable of male names either back or nasal vowels and voiced consonants, as opposed to front, non-nasal vowels and unvoiced consonants in female names. In order to test these predictions, we aggregated all of the first names spanning over the century, giving only one list of first names (e.g. ‘Marie’ was found in several decades). Only one version of phonetically equivalent names in each sex was collated (e.g. ‘Danielle’ and ‘Daniele’, homophones non-homographs, i.e. names pronounced alike but not written alike). Compound names (e.g. ‘Jean-Marie’, ‘Marie-Pierre’) were discarded as they represent a particular set of names mostly composed of a masculine name joint to a feminine name. Monosyllabic names were also discarded from the analysis because it would preclude comparing the first and last syllable. This resulted in a sample size of 275 female and 197 male popular unique names distributed across the century. A generalized linear model was then used to investigate the existence of sex-biased sound symbolic patterns in French male vs. female names. Because the response variable ‘sex’ was binary, a binomial distribution with a logit link function was specified. The explanatory variables were the articulatory features aforementioned, each repeated for the first and the stressed last syllable:

- The vowel's place of articulation – a fixed factor with three modalities (i.e. front, central or back vowel).
- The vowel's nasality – a fixed factor with two modalities (i.e. nasal and non-nasal vowel).
- Counts of voiced and unvoiced consonants (plosives and fricatives) – covariates that were standardized.

Finally, post-hoc comparisons (Tukey's range test) with a Bonferroni correction were performed for the vowel's place of articulation in order to assess comparisons between the sexes in each syllable. The general size effect was computed using Cohen's *f*^2^. A symbolic representation of the regression formula is given in the Supplementary Material (Figure S1).

#### Temporal analyses

We assessed if the potential significant sound symbolic patterns found in the previous analysis have evolved or remained constant over time between male and female French first names. Pseudo-replication was allowed but phonetically equivalent, compound and monosyllabic names were still excluded, as the aim was to study temporal variations in both the first and last syllable. This resulted in a sample size of 897 female and 790 male names distributed across all decades. To address the time series nature of the data, we first calculated all autocorrelations and partial correlations between each time lag in order to assess if the frequency of a given phonetic variable is dependent on its previous frequency. Vowel articulation was counted as the number of each type of vowel in each syllable and was centred around 0; with 0 corresponding to central vowels, 1 to front vowels and −1 to back vowels. For vowel nasality, it was counted as the proportion of each vowel type: if values are close to 0, first names contain overall fewer nasal vowels, and conversely, if values are close to 1, they contain more nasal vowels. For voiced and voiceless consonants, the mean number in each syllable was studied. Linear models were then used to describe all of the temporal trends. To study possible non-linear effects of time, we modelled a cubic and quadratic effect of decade. Sex was included as another explanatory variable and was put in interaction with time. Model comparisons using the Akaike Information Criterion were then used to assess the best describing model of the temporal variations. All statistical analyses were performed using the R software (version 3.4.4).

## Results

### Sex-biased sound symbolism

**Figure 1.**
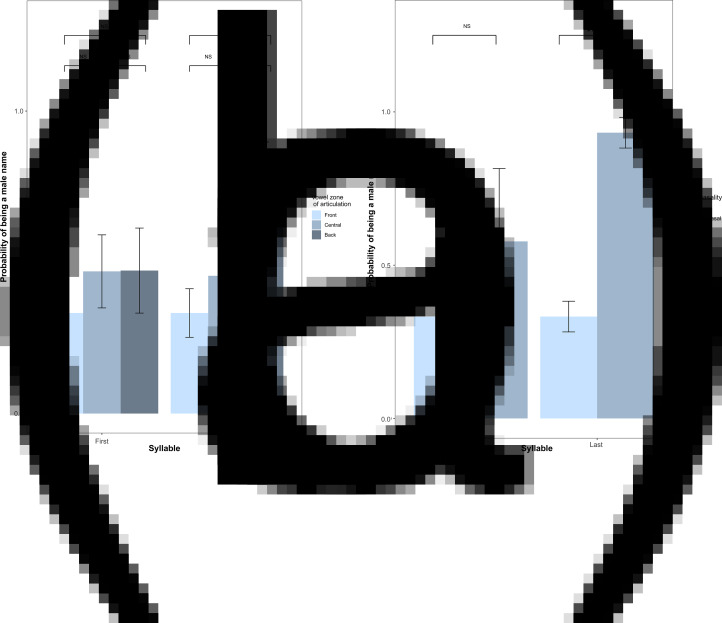
Estimates of the generalized linear model, log back-transformed to provide the probabilities of a name belonging to a male in function of the presence of a particular (a) oral vowel and (b) nasal vowel. Bars represent the mean probability associated with 95% confidence intervals. Significance code from the *post-hoc* comparisons: *** *p* < 0.001; ** *p* < 0.01; NS, non-significant.

We found a sex-biased sound structure in first names as a function of the syllable under study (Table 2). In the last stressed syllable, significant clues of masculinity were given by the vowel place of articulation (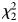 = 11.82, *p* < 0.01), nasality (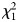 = 65.41, *p* < 0.001) and voiceless fricatives (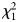 = 13.23, *p* < 0.001). Namely, male names were significantly more prone to contain back vowels like /o/ or /ɔ/ (e.g. ‘Enzo’, ‘Renaud’), instead of front or central ones such as /i/, /y/ or /a/ (respectively *t* = 1.17, *p* < 0.01; *t* = 1.35, *p* < 0.01; e.g. ‘Jackie’, ‘Luc’, ‘Bernard’). Although back vowels can be found in female names (e.g. ‘Simone’, ‘Laure’), front and central vowels are more common (e.g. ‘Emilie’, ‘Julie’, ‘Léa’, ‘Maria’) along with mid-front vowels such as /ε/ (e.g. ‘Claire’, ‘Hélène’). Male names were also significantly more likely to contain nasal vowels such as /ã/ or /ɔ̃/ (e.g. ‘Roland’, ‘Raymond’; female counter-examples, ‘Fernande’, ‘Marion’) and voiceless fricatives such as /s/ or /ʃ/ (e.g. ‘Fabrice’, ‘Michel’; female counter-examples, ‘Clemence’, ‘Blanche’). Probabilities of being a male name as a function of the type of vowel (oral and nasal) are given in Figure 1.

**Table 2.** Results of the generalized linear model. For each predictor, the estimate, standard error of the mean, the *χ*^2^, the degrees of freedom and the *p*-values associated from the likelihood ratio test of the comparison between the full model and the model without the predictor are given. For the categorical variables ‘Vowel place of articulation’ and ‘Nasality’, the estimates are given compared to the reference category (front and non-nasal vowels, respectively) for both syllables. Pseudo-*R*^2^ is the variance explained by the model (adjusted by the number of predictors) and Cohen's f^2^ the overall size effect. Significant *p*-values are in bold

Pseudo-*R*^2^ = 0.14 Cohen's *f*^2^ = 0.17 *N* total = 472 *n* female = 275 *n* male = 197	Estimate	Standard error	*χ*^2^	d.f.	*p*
Intercept	−0.69	0.18			
*First syllable*					
Vowel place of articulation			0.27	2	0.87
Central vowel	−0.12	0.25			
Back vowel	−0.11	0.29			
Nasality			0.33	1	0.56
Nasal vowel	0.31	0.54			
Voiced plosives	0.38	0.11	12.59	1	**<0.001**
Voiced fricatives	0.16	0.10	2.33	1	0.12
Voiceless plosives	0.10	0.10	0.96	1	0.32
Voiceless fricatives	−0.09	0.11	0.74	1	0.38
*Last syllable*					
Vowel place of articulation			11.82	2	**<0.01**
Central vowel	−0.18	0.24			
Back vowel	1.17	0.38			
Nasality			65.41	1	**<0.001**
Nasal vowel	2.62	0.38			
Voiced plosives	0.14	0.10	1.83	1	0.17
Voiced fricatives	0.12	0.10	0.41	1	0.23
Voiceless plosives	0.04	0.10	0.12	1	0.72
Voiceless fricatives	0.39	0.10	13.23	1	**<0.001**

Unexpectedly, in the first syllable, the probability of being a male name significantly increased as a function of the number of voiced plosives (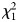 = 12.59, *p* < 0.001) such as /b/, /d/ or /g/ (e.g. ‘Bernard’, ‘Dimitri’, ‘Gustave’; female counter-examples, ‘Brigitte’, ‘Deborah’, ‘Gwenaëlle’). Within the first syllable, vowel articulation and nasality did not differ between sexes, nor did the number of voiceless fricatives (all *p* > 0.05). Eventually, articulatory features explained 14% of the variation in sex differences and the Cohen's *f*^2^ (0.17) suggests a moderate size effect (Cohen 2013).

### Temporal analyses from 1900 to 2009

**Figure 2.**
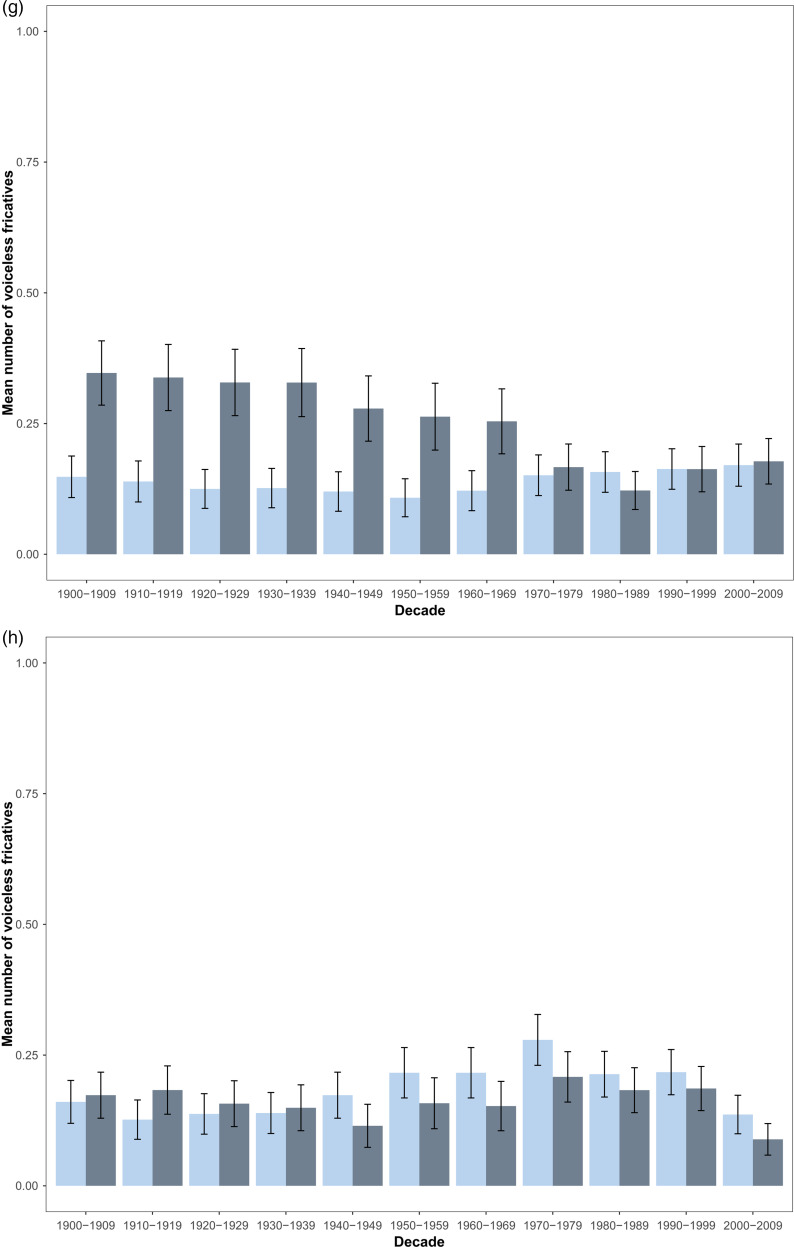
Barplots (mean ± standard-error) of the temporal variations for each decade from 1900 to 2009 of each articulatory feature that revealed significant in the sound symbolic patterns analysis. Female first names are represented in light blue and male first names in dark blue. The vowel's place of articulation is represented in (a) last syllable and (b) first syllable. Vowel's nasality in the (c) last syllable and (d) first syllable. Mean number of voiced plosives are represented in the (e) last syllable and (f) first syllable. Lastly, mean number of voiceless fricatives are represented in the (g) last syllable and (h) first syllable. Vowel articulation accounts for the number of each type of vowel in each syllable and were centred around 0; with 0 more central vowels, 1 more front vowels and −1 more back vowels. For vowel nasality, it accounts for the number of each vowel type: if values are close to 0, first names contain fewer nasal vowels, and conversely, if values are close to 1, they contain more nasal vowels.

Trends investigated were the vowel's place of articulation, vowel's nasality, the number of voiced plosives and voiceless fricatives in both the first and last syllable. All trends are shown in Figure 2.

Analyses of the autocorrelations and partial correlations revealed that the frequency of each articulatory feature at a given timepoint is mostly independent of its previous frequency (most *p* > 0.05, all autocorrelations and partial correlations are given in the Supplementary Material, Table S1).

The proportion of oral vowels across time in the last syllable of both male and female names showed a cubic change (*F*_1,1686_ = 14.01, *p* < 0.01, Figure 2a) and the overall difference in proportion between the sexes was significant (*F*_1,1686_ = 33.41, *p* < 0.001). Interestingly, female names tended to be ‘masculinized’ (i.e. contained more central and back vowels, especially the former) over time starting from the 1960s with convergent values between male and female names towards 2009. In the first syllable, no overall difference in proportion was observed between the sexes (*F*_1,1686_ = 1.62, *p* = 0.22), but both followed a quadratic temporal change (*F*_1,1686_ = 38.71, *p* < 0.001, Figure 2b). In the last syllable, the difference in proportion of names with nasal vowels was different between male and female names (*F*_1,1686_ = 117.25, *p* < 0.001) and both remained more or less constant over time (*F*_1,1686_ = 1.46, *p* = 0.24, Figure 2c). In the first syllable, a slight difference in proportion was observed (*F*_1,1686_ = 6.34, *p* < 0.05), and both sexes followed a quadratic change over time (*F*_1,1686_ = 51.59, *p* < 0.001, Figure 2d).

In the last syllable, no sex difference and no temporal change in the mean number of voiced plosives were observed (respectively *F*_1,1686_ = 1.11, *p* = 0.30; *F*_1,1686_ = 4.24, *p* = 0.054, Figure 2e). In the first syllable, overall difference in voiced plosives between the sexes was significant (*F*_1,789_ = 87.81, *p* < 0.001), but no change was observed over time (Figure 2f), although the interaction between sex and a quadratic effect of time was significant (*F*_1,1686_ = 8.48, *p* < 0.01). Overall differences in the mean number of voiceless fricatives between the sexes was found in the last syllable (*F*_1,1686_ = 60.09, *p* < 0.001). In both sexes, the mean number of voiceless fricatives followed a cubic evolution through time (*F*_1,1686_ = 12.46, *p* = 0.023, Figure 2g), and an interaction between sex and time revealed to be significant (*F*_1,1686_ = 30.66, *p* < 0.001). Lastly, the mean number of voiceless fricatives in the first syllable for both sexes linearly varied over time (*F*_1,1686_ = 31.50, *p* < 0.001, Figure 2h) and an overall difference between the sexes was observed (respectively *F*_1,1686_ = 103.32, *p* < 0.001). The interaction between sex and time was also significant (*F*_1,1686_ = 55.59, *p* < 0.001).

## Discussion

French first names exhibited sex differences in the distribution of vocalic sounds: low-frequency vowels (i.e. back and nasal) were more likely to be found in masculine names while higher frequency vowels (i.e. front and non-nasal) as well as central vowels (i.e. /a/) were more frequent in female names.

This sex-biased sound symbolism pattern was found in the last syllable, which is perceptually prominent in French, while in English, a similar sex-biased symbolism was reported for the first stressed syllable (Pitcher et al. 2013). However, regarding consonants, our results were more unexpected. Indeed, the mean number of voiceless fricatives (i.e. /f/, /s/ and /ʃ/; e.g. ‘Joseph’, ‘Alexis’, ‘Michel’) was higher in male than female names within the final stressed syllable (e.g. female names: ‘Delphine’, ‘Clarisse’). This is surprising according to the ‘frequency-code’ theory since their higher domain of frequency, relatively to voiced consonants, would rather be associated with indexical cues of smallness. The second unexpected finding was the presence of voiced plosives in the first syllable (i.e. /b/, /d/ and /g/; e.g. ‘Bernard’, ‘David’, ‘Gabriel’; e.g. female names, ‘Brigitte’, ‘Geraldine’), which is theoretically perceptually non-prominent in French. A possible explanation is that these consonantal patterns may perceptually compensate for each other, by which the presence in masculine names of voiceless fricatives in the last stressed syllable is perceptually counterbalanced by the presence of voiced consonants in the unstressed one. Otherwise, in a more general manner, vowels and consonants in the first and last syllable may be perceptually associated with different physical qualities. In this sense, while oral and nasal vowels could refer to body size, consonants might evoke other qualities such as shape or speed (Berlin 2006). For instance, it has been shown that people perceive a form as rounder if its signifier contains voiced consonants (such as /b/, /m/, /l/ or /n/) and as sharper if it contains voiceless stops (such as /k/, /p/, /t/) (Sidhu and Pexman 2015; Nielsen and Rendall 2013; Maurer et al. 2006). In the case of voiced plosives in the first syllable of male names, another explanation can be invoked as it is in accordance with results observed in American and Indian forenames (Slepian and Galinsky 2016). The authors showed a voiced gender naming effect, whereby the initial phonemes of masculine first names were voiced, as opposed to unvoiced in feminine names. They argued that voiced phonemes would sound ‘harder’ as a consequence of the vocal folds vibrating during pronunciation, whereas unvoiced phonemes will sound ‘softer’ to the ear as a consequence of unmodulated airflow, which in both cases would perceptually reinforce the stereotyped representations of males and females having respectively ‘tougher’ vs. ‘tender’ personalities and behaviours. Interestingly, the endorsement of these traditional gender stereotypes related to these ‘tougher/harder’ vs. ‘softer/tender’ dimensions moderated the influence of voiced and unvoiced phonemes on masculine vs. feminine judgments.

The name selected by parents for their offspring is, most of the time, linked to the assigned sex at birth, probably because such an information takes on great importance in both the perception of the bearer's sex properties by conspecifics in the social environment, and in the bearer's reinforcement of sexual identity and gender role (Pilcher 2016). In human societies, males and females have distinct roles and different reproductive strategies (Schmitt 2015). Owing to the associated sex-sound symbolism, giving a masculine or feminine name to conform to sex stereotypes could thus be seen as a form of parental investment with a lifelong-lasting effect. Although these effects have not been measured yet in reproductive value, it remains to be shown whether or not they influence fitness-related traits. However, the fact that most first names are sex-specific suggests that they are not fully socially neutral, and many studies have disclosed the influence of given names on some social trait, such as social desirability (Gebauer et al. 2012; Busse and Seraydarian 1978) and social outcomes (Cotton *et al.* 2008; Figlio 2005; Hodson and Olson 2005; Harari and McDavid 1973). For instance, several studies have shown that having only the information of a masculine or feminine name already influences the bearer's job and career outcomes (Kasof 1993; Moss-Racusin et al. 2012; Steinpreis et al., 1999).

Yet while our results support the idea that humans possess a cognitive bias to assign different phonetic material to either sex, the relatively small amount of variance explained in sex differences (~14%) and the relatively modest size effect (Cohen's *f*^2^ = 0.17) suggest that other factors than sexually sound symbolic patterns need to be considered when parents choose a particular name for their child. Evidence shows that the cultural environment is undeniably one of them (Acerbi and Bentley 2014; Barucca et al. 2015; Bentley et al. 2004; Berger et al. 2012; Xi et al. 2014). For instance, Bentley et al. (2004) have shown that name distributions and changes over time followed power laws, which were predicted by a simple mechanism of cultural drift and random copying between individuals, assuming that names are value-neutral in regards to fitness. Other models have been used to describe their distributions across time and space, the rate of innovation and their diversity, such as activation–inhibition processes (Zanette 2012), individual preferences and social influence (Xi et al. 2014), and spatial–temporal homogeneity (Bentley and Ormerod 2012). Most interestingly, Berger et al. (2012) have shown that names are more likely to be chosen when similar-sounding names in terms of phonetic similarity (i.e. sharing phonemes and their position within the name) have been popular the previous year, regardless of the names’ gender. For instance, their model predicted that the popularity of the name ‘Karen’ depended on popular names that possessed the same first phoneme (i.e. /k/), such as ‘Carl’ (a male name) and ‘Katie’ (a female name). Predicted popularity was also correlated with other cultural items such as hurricanes’ names (i.e. ‘Katrina’), suggesting a strong effect of other cultural items on naming processes.

In this context, the temporal variations of the articulatory features suggest a strong effect of culture, given the somewhat stochastic variations of some phonetic variants, such as the frequency of occurrence of voiced plosives and voiceless fricatives. Nonetheless, we feel that particular attention should be given to the vowel's place of articulation. Its evolution in the stressed syllable of female first names suggests that high-frequency sounds were considered as most feminine in the 1960s, a period after which we notice an increase in phonetic masculinization that continues up to 2009. For instance, names with front vowels (e.g. ‘Marie’) in the early 1900s were more frequent than those with central and back vowels (e.g. ‘Léa’, ‘Manon’), which increased in frequency in the 1960s up to the 2000s. Interestingly, an earlier study dealing with the evolution of feminization across the last century has shown that the ‘ideal’ waist-to-hip ratio (WHR), an important component of men's mate preferences, seemed to have followed the same trend in Western society. This ‘ideal’ WHR, as assessed through Playboy models and Miss pageants from 1920 to 2014, is most feminine in the 1960s (lower WHR values) then becomes less and less feminine until the 2010s (higher WHR values) (Bovet and Raymond 2015). Additionally, a meta-analysis on the self-perception of femininity and masculinity, as assessed through the Bem Sex–Role Inventory and the Personal Attributes Questionnaire, showed that American women perceived themselves as more masculine over time from the early 1970s to the mid 1990s (Twenge 1997), with additional findings demonstrating a decrease in endorsing feminine traits in women after the 2000s (Donnelly and Twenge 2017). Two other meta-analyses investigating women's own assertiveness from 1931 to 1993 showed that it decreased from 1946 to 1967, but increased from 1968 to 1993 (Twenge 2001). Such changes from the 1960s might be closely linked to historical political feminists’ movements particularly active in this era during which awareness of inequalities in civil rights and social positions has been increasing. We hypothesize that one possible strategy to compensate for such inequalities is to masculinize some traits in women in order to compete against men for the same rights and privileges, at least in industrialized and traditionally male-dominated societies.

## Conclusions

Overall, the present study offers some promising opportunities for follow-up studies that would lead to a better understanding of naming processes. An interesting avenue for further research would be to model the relative importance of different selective pressures (sexual and cultural, or a joint effect) acting on the phonetic dimorphism, names’ frequency and the emergence of novel names. Most importantly, to fully acknowledge the action of sexual selection on the phonetic dimorphism, a study on names and their relationship to reproductive value is required. One limitation is that not all names from each decade were analysed and a particular attention should be given to rare names in order to strengthen the present results. Moreover, particular attention should also be given to syllables between the first and last ones, as they can potentially play a particular role. Further inquiries in sound symbolic patterns in first names in dead and modern languages should be performed, so as to find some universal components in vowel quality to convey perceived masculinity and femininity.

## Supplementary material

For supplementary material accompanying this paper visit http://dx.doi.org/10.1017/ehs.2019.7. click here to view supplementary material

## Data access

The data and the R code are available at https://figshare.com/s/5618e367fae4c5774272
