# Supplementary material for: Sex-biased sound symbolism in French first names
Source: Evol Hum Sci. 2019 Jul 12;1:e7. doi: 10.1017/ehs.2019.7 (PMC10427910; doi:10.1017/ehs.2019.7)
Supplement: Supplementary file 1 [file S2513843X19000070sup001.docx]

Figure S1. Symbolic representation of the generalized linear model used to study sex-bias sound symbolic patterns.

$g\left( Y_{ij} \right)={}_{0}+{}_{1}X_{ij}+ {}_{2}X_{ij}+ {}_{3}X_{ij}+{}_{4}X_{ij}+{}_{5}X_{ij}+{}_{6}X_{ij}+{}_{7}X_{ij}+{}_{8}X_{ij}+ {}_{9}X_{ij}+ {}_{9}X_{ij}+ {}_{10}X_{ij}+ {}_{11}X_{ij}+{}_{12}X_{ij}+ e_{ij}$

With i = first name *i* and j = first name sex

g – logit link function

${}_{0}$ – intercept

${}_{1}$– vowel height of the first syllable (fixed factor)

${}_{2}$ – vowel nasality of the first syllable (fixed factor)

${}_{3}$ – voiced plosives of the first syllable (covariate)

${}_{4}$ – voiced fricatives of the first syllable (covariate)

${}_{5}$ – voiceless plosives of the first syllable (covariate)

${}_{6}$ – voiceless fricatives of the first syllable (covariate)

${}_{7}$– vowel height of the last syllable (fixed factor)

${}_{8}$ – vowel nasality of the last syllable (fixed factor)

${}_{9}$ – voiced plosives of the last syllable (covariate)

${}_{10}$ – voiced fricatives of the last syllable (covariate)

${}_{11}$ – voiceless plosives of the last syllable (covariate)

${}_{12}$ – voiceless fricatives of the last syllable (covariate)

$e_{ij}$ – error component

Table S1. Autocorrelations and partial correlations (in italics) calculated for each sex (F = female; M = male) for each phonetic variable at each time lag. S1 and S2 respectively correspond to the first and last syllable. Each time lag consecutively corresponds to the period of time between each decade. For instance, lag 1 refers to the period of time between the first two decades (1900-1909 and 1910-1919), lag 2 to the second and third decade (1910-1919 and 1920-1929), so on and so forth. Autocorrelations refer to the correlations between two consecutive time lags. Partial correlations refer to the correlations between two consecutive time lags while controlling for the correlations at all other time lags. Autocorrelations at lag 0 is 1. Significant p values at the 0.05 level are in bold.

| **Phonetic variable** | **Sex** | **Lag 1** | **Lag 2** | **Lag 3** | **Lag 4** | **Lag 5** | **Lag 6** | **Lag 7** | **Lag 8** | **Lag 9** | **Lag**  **10** |
| --- | --- | --- | --- | --- | --- | --- | --- | --- | --- | --- | --- |
| **Vowel articulation (S1)** | F | 0.40  *0.40* | 0.28  *0.15* | -0.02  *-0.21* | 0.05  *0.10* | -0.10  *-0.10* | -0.14  *-0.14* | -0.08  *0.10* | -0.36  *-0.43* | -0.30  *-0.08* | -0.23  *0.16* |
|  | M | 0.46  *0.46* | 0.21  *-0.002* | 0.14  *0.06* | 0.10  *0.02* | -0.26  *-0.41* | -0.30  *-0.06* | -0.18  *0.05* | -0.16  *-0.07* | -0.30  *-0.16* | -0.19  *-0.04* |
| **Vowel articulation (S2)** | F | 0.55  *0.55* | 0.12  *-0.26* | 0.10  *-0.05* | 0.20  *-0.12* | 0.23  *-0.10* | 0.20  *-0.07* | 0.11  *0.005* | 0.12  *-0.18* | 0.13  *-0.05* | 0.08  *-0.04* |
|  | M | 0.51  *0.51* | -0.03  *-0.39* | -0.24  *-0.03* | -0.36  *-0.29* | -0.43  *-0.25* | -0.23  *0.02* | -0.07  *-0.27* | 0.07  *0.04* | 0.18  *-0.09* | 0.11  *-0.24* |
| **Nasality (S1)** | F | 0.57  *0.57* | 0.34  *0.03* | 0.10  *-0.16* | 0.008  *-0.003* | -0.06  *-0.03* | -0.26  *-0.29* | -0.30  *-0.06* | -0.42  *-0.21* | -0.30  *0.03* | -0.17  *0.08* |
|  | M | 0.42  *0.42* | 0.17  *-0.006* | -0.01  *-0.10* | 0.04  *0.11* | -0.02  *-0.07* | -0.13  *-0.16* | -0.31  *-0.22* | -0.33  *-0.15* | -0.22  *-0.02* | -0.10  *-0.007* |
| **Nasality (S2)** | F | 0.38  *0.38* | 0.26  *0.14* | 0.20  *0.08* | -0.10  *-0.27* | -0.42  *-0.45* | -0.27  *-0.002* | -0.23  *0.14* | -0.20  *0.10* | -0.10  *-0.14* | -0.006  *-0.25* |
|  | M | **0.68**  ***0.68*** | 0.14  ***-0.60*** | -0.34  *-0.26* | -0.56  *-0.04* | -0.45  *0.03* | -0.26  *-0.33* | -0.02  *0.08* | 0.11  *-0.19* | 0.14  *-0.03* | 0.07  *-0.23* |
| **Voiced plosives (S1)** | F | 0.22  *0.22* | 0.25  *0.21* | -0.27  *-0.40* | -0.35  *-0.35* | -0.34  *-0.06* | -0.23  *-0.06* | -0.07  *-0.14* | 0.10  *-0.03* | 0.06  *-0.16* | 0.13  *-0.10* |
|  | M | **0.70**  ***0.70*** | 0.46  *-0.07* | 0.05  *-0.49* | -0.24  *-0.19* | -0.37  *0.17* | -0.49  *-0.29* | -0.34  *0.14* | -0.21  *0.10* | -0.06  *-0.26* | -0.001  *-0.24* |
| **Voiced plosives (S2)** | F | 0.56  *0.56* | -0.04  *-0.53* | -0.38  *-0.08* | -0.39  *-0.11* | -0.28  *-0.19* | -0.20  *-0.23* | -0.09  *-0.04* | 0.05  *-0.09* | 0.16  *-0.06* | 0.12  *-0.20* |
|  | M | 0.37  *0.37* | 0.09  *-0.05* | -0.18  *-0.23* | -0.07  *0.10* | 0.10  *0.15* | 0.11  *-0.04* | -0.02  *-0.11* | -0.34  *-0.31* | -0.34  *-0.10* | -0.21  *-0.03* |
| **Voiceless fric (S1)** | F | 0.53  *0.53* | 0.35  *0.09* | -0.20  *-0.59* | -0.34  *-0.11* | -0.43  *0.13* | -0.36  *-0.25* | -0.14  *0.06* | -0.02  *0.02* | 0.08  *-0.28* | 0.04  *-0.06* |
|  | M | 0.03  *0.03* | -0.04  *-0.04* | **-0.60**  ***-0.60*** | -0.15  *-0.19* | -0.14  *-0.30* | 0.33  *-0.15* | 0.16  *-0.12* | 0.09  *-0.27* | -0.11  *-0.10* | -0.08  *-0.11* |
| **Voiceless fric (S2)** | F | **0.67**  ***0.67*** | 0.25  *-0.35* | -0.07  *-0.12* | -0.42  *-0.44* | -0.51  *0.08* | -0.32  *0.14* | -0.15  *-0.14* | -0.07  *-0.26* | 0.05  *0.01* | 0.07  *-0.03* |
|  | M | **0.78**  ***0.78*** | 0.49  *-0.31* | 0.19  *-0.19* | -0.11  *-0.23* | -0.27  *0.11* | -0.39  *-0.20* | -0.47  *-0.21* | -0.38  *0.24* | -0.22  *0.08* | -0.10  *-0.25* |
